# Trends of follow-up clinic visits and admissions three-months before and during COVID-19 pandemic at Tikur Anbessa specialized hospital, Addis Ababa, Ethiopia: an interrupted time series analysis

**DOI:** 10.1186/s12913-021-06730-8

**Published:** 2021-07-23

**Authors:** Workeabeba Abebe, Alemayehu Worku, Tamirat Moges, Nuhamin Tekle, Wondowossen Amogne, Tewodros Haile, Desalew Mekonen, Abebe Habtamu, Wakgari Deressa

**Affiliations:** 1 Department of Pediatrics and Child Health, College of Health Sciences, Addis Ababa University, Addis Ababa, Ethiopia; 2 School of Public Health, College of Health Sciences, Addis Ababa University, Addis Ababa, Ethiopia; 3 Department of Family Medicine, College of Health Sciences, Addis Ababa University, Addis Ababa, Ethiopia; 4 Department of Internal Medicine, College of Health Sciences, Addis Ababa University, Addis Ababa, Ethiopia

**Keywords:** Admission, COVID-19, Ethiopia, Follow-up visit, Healthcare utilization, Trends

## Abstract

**Background:**

Following the first report of the COVID-19 case in Ethiopia on March 13, 2020, the country promptly adopted a lockdown policy to contain the virus’s spread. Responding to the healthcare burden imposed by the COVID-19 pandemic had to be coupled with ensuring essential health care services. This study assessed the impact of COVID-19 on the trends in hospital visits and admissions at Tikur Anbessa Specialized Hospital by comparing the rate of follow-up clinic visits and admissions for the 3 months before and after the first report of the COVID-19 case.

**Methods:**

A retrospective, time-series study examined the trend in follow-up visits and admissions between December 11, 2019, to June 7, 2020, with the 1st case of the COVID-19 report in Ethiopia (March 13, 2020) as a reference time. To control seasonal effects and random fluctuation, we have compared health care utilization to its equivalent period in 2018/19. A data extraction tool was used to collect secondary data from each unit’s electronic medical recordings and logbooks.

**Results:**

A total of 7717 visits from eight follow-up clinics and 3310 admissions were collected 3 months before the onset of COVID-19. During the following 3 months after the onset of the pandemic, 4597 visits and 2383 admissions were collected. Overall, a 40.4% decrease in follow-up visits and a 28% decline in admissions were observed during the COVID-19 pandemic. A drop in the daily follow-up visits was observed for both genders. The number of visits in all follow-up clinics in 2019/2020 decreased compared to the same months in 2018/19 (*p* < 0.05). Follow-up visits were substantially lower for renal patients (− 68%), patients with neurologic problems (− 53.9%), antiretroviral treatment clinics (− 52.3%), cardiac patients (− 51.4%). Although pediatric emergency admission was significantly lower (− 54.1%) from the baseline (*p* = 0.04), admissions from the general pediatric and adult wards did not show a significant difference.

**Conclusions:**

A decline in follow-up clinic visits and emergency admissions was observed during the first months of the COVID-19 pandemic. This will increase the possibility of avoidable morbidity and mortality due to non-COVID-19-related illnesses. Further studies are needed to explore the reasons for the decline and track the pandemic’s long-term effects among non-COVID-19 patients.

## Background

The coronavirus disease 2019 (COVID-19) pandemic continues to challenge the healthcare systems around the world. Responding to the healthcare burden imposed by the pandemic required intense resources. Even more, this had to be coupled with ensuring continued access to essential healthcare services [1]. Countries struggled to allocate resources for these needs fairly. Hospitals operated with less capacity due to the distribution of resources for COVID-19 prevention, detection, and case management. Some hospitals’ care, such as elective surgery and other non-critical medical services, were purposefully curtailed [2]. In addition, following the initial public health messaging that discouraged unnecessary healthcare use, many patients delayed or canceled follow-up appointments in hospitals [3, 4]. In Singapore, doctor visits decreased by up to 30%, while a massive drop of 63.8% in pediatric emergency healthcare utilization was observed in Germany, and a comparable 63.5% reduction in emergency department visits was reported in the USA [5–7]. The delayed access or provision of care affected acute and chronic care [8, 9] and increased adverse outcomes from non-COVID-19-related illnesses [10, 11].

Reports from Middle East, North Africa, and West Asia region also showed that essential treatments, including chemotherapy, surgery, and radiation therapy, were delayed in 29 to 44% of centers [12]. Restriction of acceptance of new patients and clinical care delivery was reported as negatively affected in one-third of centers [12]. In Africa, health service for vulnerable populations like people living with HIV was also believed to be significantly affected. Reduction in patient volume means less identification of index patients through clinic-based testing, and reduction in counseling could affect the initiation and retention of antiretroviraltreatment (ART) [13].

In Ethiopia, the first case of COVID-19 was reported on March 13, 2020 [14]. The country promptly adopted a lockdown policy to contain the spread of the virus, schools were closed on March 16, 2020, and international travel bans were put in place on March 20, 2020 [14]. Subsequently, stay at home order was recommended to the public and a state of emergency was declared on April 8, 2020 [14, 15]. Despite efforts to prevent the spread of COVID-19 in Ethiopia, new cases continued to emerge among individuals with a travel history and even without clear exposure indicating the emergence of community transmission. Soon after, the presence of community transmission has already been established, and the virus has been reported from all corners of the country. As of June 11, 2021, the total number of confirmed COVID-19 cases in Ethiopia was 272,914, of which 4209 died [16].

Ethiopia reported fewer cases than other countries that had a significant surge of COVID-19 cases in the first months of 2020. However, to prevent the spread of the COVID-19 virus in hospitals and implement effective case management, hospitals were designated as COVID-19 and non-COVID-19 case management centers [17]. Patients suspected of having COVID-19 were put in an isolation ward, and those whose tests confirmed the presence of COVID-19 were transferred out to COVID-19 centers. Although Tikur Anbsaa Specialized Hospital (TASH) was among the non-COVID-19 case management centers, a sharp drop in the attendance of follow-up and emergency visits was observed.

We hypothesized that many patients with medical illnesses did not seek hospital care due to uncertainty of the pandemic, lockdown policy, and other restrictions imposed during the initial phase of the pandemic. Therefore, this study assessed the impact of COVID-19 on the trends of non-COVID follow-up visits and admissions at Tikur Anbessa Specialized Hospital, Addis Ababa, Ethiopia. In addition, a better understanding of the impact of COVID-19 on medical admissions over time would provide insight for policymakers and health care planners.

## Methods

### Setting

This study was conducted at Tikur Anbessa Specialized Hospital (TASH), a health professionals training center located in Addis Ababa, Ethiopia. The hospital serves a large population of patients referred for outpatient and inpatient care in all core specialties (internal medicine, emergency, critical care, surgery, pediatrics, and obstetrics/gynecology). TASH has about 700 beds; about 150 are medical, and 172 beds are in pediatrics. Clinical care teams composed of nursing staff, medical interns, and residents supervised by attending staff provide all care for patients.

### Study design and period

A retrospective time-series study was conducted to collect daily follow-up visits and monthly admissions data at TASH between December 11, 2019, and June 9, 2020. A before-after study was done to analyze the trend in daily follow-up visits and monthly admissions, with the 1st case of the COVID-19 report (March 13, 2020) as a cut of point. To control seasonal effects and random fluctuation, we compared health care utilization to its equivalent period in 2018/19. The hospital used the Ethiopian calendar (EC), in which all months have equal size (30 days) except the 13^th^month, usually 5–6 days. Table 1 shows the study period in the Ethiopian calendar with its corresponding Gregorian calendar. The data extraction took place during the second half of June 2020.

**Table 1:** Showing Ethiopian Calendar with its corresponding Gregorian Calendar used during data collection and presentation

Months represented in the table/figure	Ethiopian Calendar	Exact date of Collection from Gregorian Calendar
December	*Tahsas* 1–30	December 11 – January 9
January	*Tirr* 1–30	January 10 – February 8
February	Yekatit 1 - *Megabit 3*	February 9 – March 12
March	*Megabit* 4^a^ –30	March 13^a^ – April 8
April	*Miazia* 1–30	April 9 – May 8
May	Ginbot 1–30	May 9 – June 9

^a^The date when the first COVID-19 case was reported in Ethiopia

### Study participants

In this study, we assessed the impact of COVID-19 on the trends in hospital visits and admissions at TASH in Ethiopia. First, we compared the rate of follow-up clinic visits and admissions for the 3 months before and after the first report of the COVID-19 case in 2020. In addition, we compared the trends in hospital visits to the equivalent period in the previous year (Dec 2018 to May 2019).

### Data sources

We collected secondary data during the selected periods from the hospital’s Electronic Medical Recording (EMR) and logbooks in each outpatient clinic, emergency/casualty department, and wards of TASH. TASH introduced an EMR system for most adult clinics in 2018; however, the health records from pediatrics and some clinics from the adult side were still paper-based recordings.

### Variables

A study team comprised of clinicians and public health experts developed a data extraction tool to collect data on sex (male, female), age (under 5 years, 5–12 years, 13–44 years, 45–64 years, 65 years, and over) daily outpatient visits and inpatient numbers. We also used the following specialty follow-up clinics: neurology, cardiac, hematology-oncology, pediatric ART, endocrine, high-risk infant clinic, renal and gastrointestinal. We did not obtain individual data; it was either daily/monthly data disaggregated by age and sex, which were well documented on the logbook/hospital database. The primary outcome variables were daily follow-up visits and monthly hospital admissions of adult and pediatric wards.

### Inclusion/exclusion

Follow-up clinic visits, and admissions, between December 11, 2019, and June 9, 2020, as well as their equivalents in the previous year follow-up clinic visits and admissions, were included in this study. Clinics with incomplete records of some follow-up visits during the study period were excluded.

### Data collection and procedures

A one-day training was given to data collectors on the data collection tools and procedures. Trained nurses from each unit collected data that were collected as part of the routine hospital service. Eight outpatient follow-up clinics (four adult and four pediatric clinics) were included. These clinics were selected based on the availability of the documentation of the required information. In addition, both adult and pediatric inpatient wards were included.

### Statistical analysis

All data were entered into a password-secured database using Statistical Package for the Social Sciences (SPSS) version 24. After cleaning, the data were exported to STATA version 14. We analyzed the trend of daily follow-up visits and monthly hospital admissions. We compared the health care utilization data with equitant data from the previous year (2018/2019) to control seasonal effects and random fluctuation; no statistical method was used to control these effects. Data were summarized using graphs and mean (standard error of the mean [SEM]). Wilcoxon Signed-Ranks Test was used to detect mean differences between the 2018/19 and 2019/20 trends of the follow-up visits and ward admission differences. A *P-value* below 0.05 was considered significant.

## Results

A total of 12,314 follow-up visits and 5693 hospital admissions data were collected, all non-COVID-19. Taking the first case of COVID-19 report in the country as a cut of, 7717 follow-up visits happened before (3 months) and the rest, 4597 follow-up visits, after (3 months). Separately, 3310 hospital admissions before (3 months) and 2383 admissions after (3 months) of the 1st case of the COVID-19 report were collected (Table 2). Similarly, 15, 583 follow-up visits and 6639 hospital admissions data of the preceding year of the same time were also collected. The decrease in follow-up clinic visits and admissions after the onset of COVID-19 was 40.4% and 28%, respectively. We observed variations in the number of visits and admissions across the follow-up clinics and inpatient wards. Follow-up visits were substantially lower for renal patients (− 68% below baseline), patients with neurologic problems (− 53.9%), Antiretroviral treatment clinics (− 52.3%), cardiac patients (− 51.4%t). Although pediatric emergency admission was significantly lower (− 54.1%) from the baseline, admissions from the general pediatric (− 14.7%) and adult (− 6.9%) wards were not that much affected**.**

**Table 2:** Number of follow-up visits and admissions before and during COVID-19, and percentage reduction, TASH, 2020

Characteristics	Pre-COVID-19			Total	During-COVID-19			Total	^a^Percent (%) reduction
	Dec	Jan	Feb		Mar	Apr	May		
**Follow-up clinics**									
Neurology	599	643	467	1709	275	265	247	787	− 53.9
Cardiac	511	618	422	1551	288	260	206	754	−51.4
Hema. - Oncology	481	525	582	1588	449	460	465	1374	−13.5
Pediatric - ART	296	337	264	897	127	152	149	428	−52.3
Endocrine	251	238	269	758	222	205	139	566	−25.3
HRIC	226	110	196	532	125	130	133	388	−27.1
Renal	123	128	124	375	38	40	42	120	−68.0
Gastro-intestinal	110	71	126	307	88	58	34	180	− 41.4
**2019/20 (Overall visit) Total**	**2597**	**2670**	**2450**	**7717**	**1612**	**1570**	**1415**	**4597**	**−40.4**
2019/20 (Ped. ER admission)	406	421	398	1225	200	212	150	562	−54.1
2019/20 (Ped. Ward admission)	532	535	469	1536	460	480	370	1310	−14.7
2019/20 (Adult ward admission)	173	181	195	549	185	164	162	511	−6.9
**Overall admission**	**1111**	**1137**	**1062**	**3310**	**845**	**856**	**682**	**2383**	**−28.0**

^a^Percentage Changes in follow-up clinics visits & admission

We described the daily mean and standard error of mean separately for males and females who visited outpatient clinics 3 months before and after the first report of Ethiopia’s COVID-19 case. In total, 131 observations (daily hospital follow-up visits in 6 months) occurred at TASH during the analyzed period, with 66 before and 65 after the first COVID-19 case seen in Ethiopia. There were statistically significant declines in the number of daily visits in all follow-up clinics except the Endocrine and GI clinics (Table 3).

**Table 3:** Comparison of the number of outpatient visits by gender before and after the first case of COVID-19, TASH, 2020

Clinics	Average daily visits 3 months before 1st COVID case		Average daily visits 3 months after 1st COVID case.		***P***-value
	Daily mean (±SEM)	95% CI	Daily mean (±SEM)	95% CI	
^a^ **ART clinic**					
Male	13.8 (0.7)	12.3–15.2	12.2 (0.8)	10.5–13.8	0.07
Female	23.6 (1.0)	21.5–25.6	19.7 (1.3)	17.1–22.3	0.01
**Cardiac clinic**					
Male	24.5 (2.4)	19.8–29.3	11.8 (1.7)	8.3–15.2	0.00
Female	37.2 (3.6)	30.0–44.6	17.1 (2.4)	12.3–21.8	0.00
**Chest clinic**					
Male	13.2 (1.5)	10.1–16.2	7.0 (1.0)	5.0–9.0	0.00
Female	12.8 (1.6)	9.6–15.9	8.5 (1.3)	6.0–11.0	0.02
**Endocrine clinic**					
Male	19.0 (3.1)	12.7–25.3	15.1 (2.5)	9.9–20.1	0.16
Female	23.9 (3.9)	16.0–31.7	17.5 (2.9)	11.6–23.4	0.09
^**b**^ **GI clinic**					
Male	2.0 (0.4)	1.3–2.8	1.5 (0.3)	0.8–2.1	0.13
Female	1.2 (0.2)	0.8–1.7	0.7 (0.2)	0.4–1.1	0.05
^**c**^ **HRIC clinic**					
Male	3.3 (0.2)	2.9–3.8	2.0 (0.2)	1.7–2.3	0.00
Female	2.8 (0.2)	2.3–3.2	1.9 (0.2)	1.6–2.4	0.00
**Neurology clinic**					
Male	18.7 (1.0)	16.7–20.7	10.7 (0.7)	9.3–12.1	0.00
Female	11.8 (0.7)	10.5–13.2	6.2 (0.4)	5.3–7.0	0.00

^a^*ART* Anti Retroviral Treatment, ^b^*GI* Gastro-Intestinal, ^c^*HRIC* High Risk Infant Clinic

**Fig. 1:**
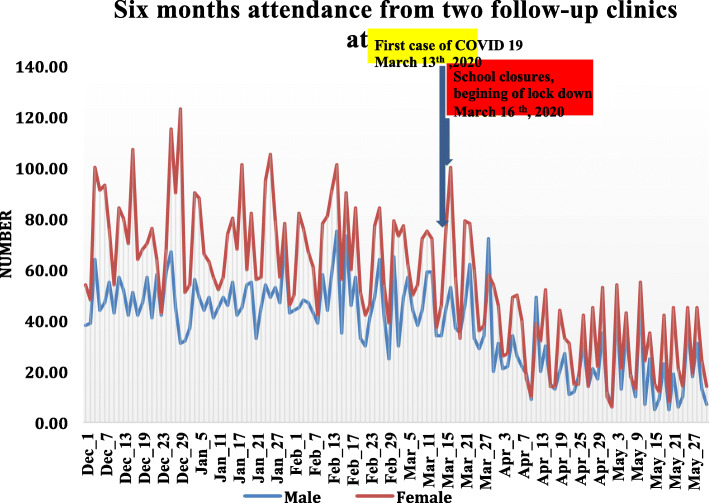
Trends in the number of daily follow-up visits from selected clinics by gender from December 2019 to May 2020

The trend for six-month daily attendance by male and female patients from two follow-up clinics (Cardiac and ART) at TASH is illustrated in Fig. 1. The clinics were selected since they had daily follow-up service throughout the week over the observation period. There was a sharp drop in the daily hospital visits for both genders in mid-March after the first COVID-19 case was reported. The hospital visits did not return to the baseline observation period by the end of May 2020.

**Fig. 2:**
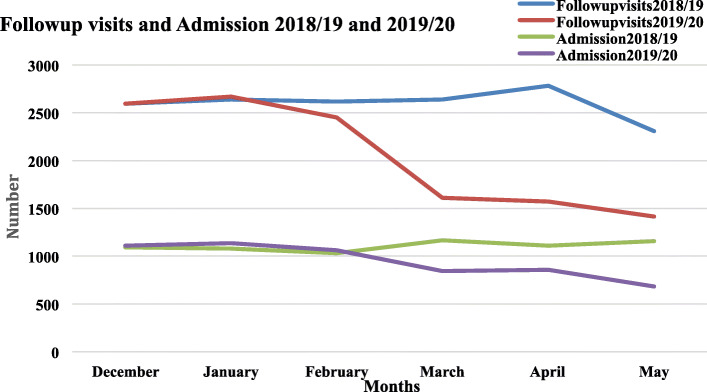
Trends in the number of daily follow-up visits from selected clinics by gender from December 2019 to May 2020

Figure 2 shows the total number of follow-up visits and admissions for 6 months of Dec 2019-May 2020 compared to last year’s same period (2018/19). The number of visits to the follow-up clinics decreased compared to the same period in 2018/19. Ward admissions at TASH decreased after February; however, the trend is not similar to the follow-up clinics.

**Fig. 3:**
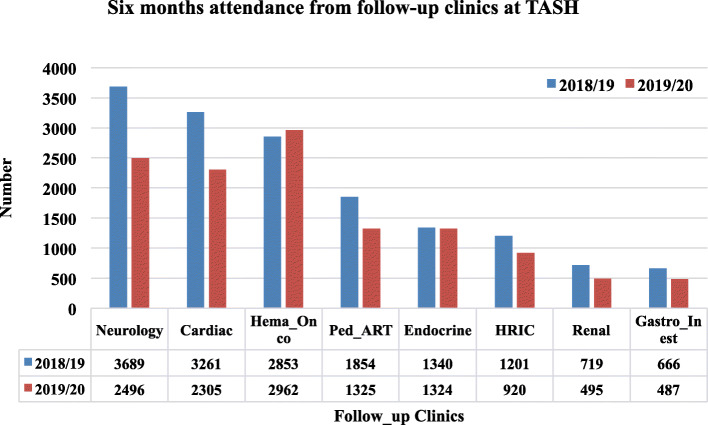
Monthly follow-up visits, December 2019 to May 2020 relative to the equivalent period in 2018/19

Figure 3 shows follow-up clinic visits separately. In 2019/20, the number of visits at the follow-up clinics decreased compared to the same period in 2018/19. The relative reduction in monthly follow-up visits was observed in all clinics, except endocrine and hematology-oncology clinics.

**Fig. 4:**
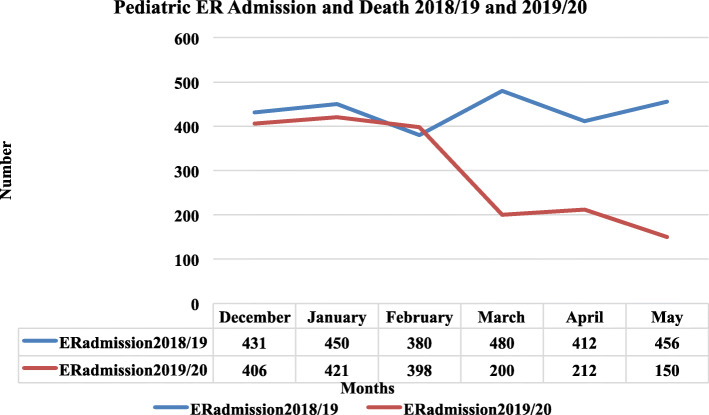
Pediatric ER Admission, December 2019 to May 2020 relative to the equivalent period in 2018/19

The trend of admissions in the pediatric emergency between December 1, 2019, and May 31, 2020, is illustrated in Fig. 4. The monthly report of the number of children who were admitted to the pediatrics Emergency room (ER) showed a significant drop in March, and there was no rebound in May (*p* = 0.04).

**Fig. 5:**
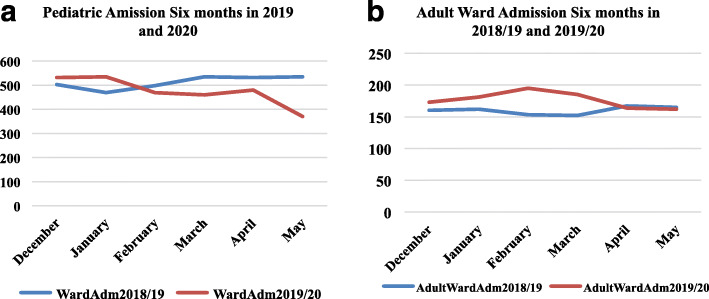
**a** and **b** Pediatrics and adult ward admissions, December 2019 to May 2020 relative to the equivalent period in 2018/19

Figure 5a and b illustrate the monthly pediatrics and adult ward admissions and deaths, respectively. Pediatrics ward admissions at TASH decreased since February, with the highest drop observed in May 2020. However, the difference was not statistically significant (*p* = 0.34). Adult medical admissions did not show any difference in 2020 compared to the previous year (*P* = 0.12).

## Discussion

This study was conducted 3 months after the 1st case of COVID-19 was reported in Ethiopia in Tikur Anbessa Specialized Hospital (TASH), the largest public hospital found in Addis Ababa, the capital city most affected by the pandemic in Ethiopia. The study demonstrated a significant decrease in follow-up clinic visits and emergency admissions during the first 3 months of the COVID-19 pandemic in the tertiary care center. Furthermore, the case numbers in follow-up clinics and emergency admissions during the observed period decreased compared to the equivalent period in the preceding year of 2018/19. The emergency admission significantly declined in the study period; however, the ward admission rate did not significantly decline compared to outpatient follow-up. The decline in the number of overall visits was consistent with the reports from a study in the USA [18]. Similar studies showed that the decrease was observed in primary health care and emergency department visits [11, 19]. These findings were valid for pediatric and adult populations across specialty clinics [20–22]. Moreover, reports from South Africa also showed a drastic decrease in outpatient visits [23] and a substantial decrease in the number of patients presenting to the emergency care during the lockdown period due to COVID 19 [24].

In a study conducted in TASH before the COVID 19 pandemic, overcrowding was one problem identified hindering the provision of quality care and care within a reasonable time [25], which might have an implication on implementation of social distancing, which is one of the preventive measures during COVID 19 pandemic [20, 26]. Studies conducted during the early stages of COVID in Ethiopia also revealed the problem of inadequate personal protective equipment in major hospitals of Addis Ababa, including TASH [38]. The overcrowding and the limited availability of personal protective equipment at TASH justifiably increase the fear of acquiring COVID 19 in this setting, which could decline follow-up visits. Reports from elsewhere documented this as an important reason for the drop in hospital visits [2, 7, 28].

The communication of alarming medical information to the public and the announcement of stay-at-home orders has also impacted healthcare-seeking behavior. Being a referral hospital, it was believed that patients who were on follow-up and those visiting the TASH emergency department often have a real need that requires hospital management. In such situations, people might have opted for a conservative, ‘watch and wait’ approach [7], while some might have resorted to traditional medicine [29], as it is a common alternative in Ethiopia. The hospital started to use telephone consultation as alternative means to treat follow-up patients during the last week of May, so we believe this would have minimal effect during the study period [30]. The hospital has no outreach services; however, patients might have received care at nearby facilities, which we have not evaluated in this study. A compelling contrary argument for the decreased hospital visit was a reduced incidence of illnesses such as community-acquired pneumonia, which was a reason for most pediatric emergency visits and in adult patients with co-morbidities [22, 31].

The delay in seeking appropriate health care is believed to have deleterious health outcomes [6] such as negatively affect people living with HIV: decrease in HIV testing and ART initiation [32] and decrease ART adherence [33], higher incidence of worsening of symptoms in diabetics patients [34], out of hospital mortality due to neoplasm, cardiac, nutritional or endocrine problems [34]. However, an increase in mortality was not observed during the study period in Ethiopia [15], and we believe that mortality may not be a reason for the decline in health service utilization [35].

The number of children admitted to the pediatric and adult wards showed − 28, which is lower with the report from the USA and Scotland [18, 36, 37]. Although the admission rate did not significantly decline compared to outpatient follow-up, we will not undermine the decline in a hospital that used to be full with patients waiting to be admitted at any time of the year. In addition, the emergency admission significantly decreased in the study period, which raised the concern of increased morbidity and mortality due to delay or lack of care [38].

The impact of COVID-19 on health care resources is enormous globally and more so in low-income countries. Ethiopia being among the low-income countries, struggles to adequately allocate human resources, support community-based testing capacity, and provide the equipment needed to manage COVID- 19 cases. In addition, it is wise to consider that the attempt to respond to the COVID-19 pandemic has compromised the attention given for non-COVID illnesses, which might be a reason for admission declines to reserve beds for possible anticipated surges of COVID 19 cases [13].

## Limitations

The paper assessed the impact of COVID-19 on the trends of non-COVID follow-up visits and admissions in Ethiopian settings. Indeed, the approach has some limitations, including the use of secondary data, and the data were not crosschecked. We took data from two departments only (pediatrics and internal medicine) and not from individuals; therefore, we did not do subgroup analysis, and consequently, it was not possible to confidently evaluate the predictors of the adverse outcomes observed. The hospital data was not collected for scientific purposes, and poor documentation was the reality in our setting, so there may be cases that were not documented (missed). TASH is a referral hospital, and the service was structured according to sub-specialty/specialty clinics, so these findings might not be generalized to primary health care and other health care settings.

## Conclusions

A significant decrease in follow-up clinics visit and emergency admissions was observed during the first months of the COVID19 pandemic. Although it is difficult to undermine the decline in a hospital admission that used to be packed with patients, the ward admission rate did not significantly decline compared to outpatient follow-up. Further studies are needed to explore the reasons for the decline and track the pandemic’s long-term effects among non-COVID 19 patients. Appropriate public health guidance on how best to access care, emphasizing the importance of visiting the healthcare facilities for severe illnesses, is required.

## Data Availability

The datasets used and/or analyzed during the current study are available from the corresponding author on reasonable request.
